# A phase II study evaluating pharmacokinetics and dosimetry of ^177^Lu-PSMA-617 radioligand therapy in Chinese participants with progressive metastatic castration-resistant prostate cancer

**DOI:** 10.1186/s13550-026-01401-3

**Published:** 2026-03-01

**Authors:** Hongcheng Shi, Jianming Guo, Wei Fan, Yonghong Li, Rui Huang, Qiang Dong, Zhi Yang, Yong Yang, Xinlu Wang, Di Gu, Li Huo, Zhigang Ji, Feng Wang, Danfeng Xu, Zhiqiang Jiang, Haifu Li, Jialu Li, Runqin Li, Luyuan Qi, Jing Wang, Dingwei Ye

**Affiliations:** 1 Department of Nuclear Medicine, Fudan University Zhongshan Hospital, Shanghai, China; 2 Department of Urology, Fudan University Zhongshan Hospital, Shanghai, China; 3 Department of Nuclear Medicine, Sun Yat-sen University Cancer Center, Guangzhou, China; 4 Department of Urology, Sun Yat-sen University Cancer Center, Guangzhou, China; 5 Department of Nuclear Medicine, West China Hospital, Sichuan University, Chengdu, China; 6 Department of Urology, West China Hospital, Sichuan University, Chengdu, China; 7 Department of Nuclear Medicine, Peking University Cancer Hospital, Beijing, China; 8 Department of Urology, Peking University Cancer Hospital, Beijing, China; 9 Department of Nuclear Medicine, The First Affiliated Hospital of Guangzhou Medical University, Guangzhou, China; 10 Department of Urology, The First Affiliated Hospital of Guangzhou Medical University, Guangzhou, China; 11 Department of Nuclear Medicine, Peking Union Medical College Hospital, Beijing, China; 12 Department of Urology, Peking Union Medical College Hospital, Beijing, China; 13 Department of Nuclear Medicine, Nanjing First Hospital, Nanjing, China; 14 Department of Urology, Ruijin Hospital, Shanghai Jiao Tong University School of Medicine, Shanghai, China; 15 Novartis Biomedical Research, Shanghai, China; 16 Novartis Development, Beijing, China; 17 Novartis Development, Shanghai, China; 18 Novartis Pharmaceuticals Corporation, Shanghai, China; 19 Novartis Pharmaceuticals AG, Basel, Switzerland; 20 Department of Nuclear Medicine, Xijing Hospital, 127 Changle W Rd, Xincheng, Xi’an, Shaanxi, 710033 China; 21 Department of Urology, Fudan University Shanghai Cancer Center, 270 Dongan Rd, 270, Xuhui District, Shanghai, 200032 China

**Keywords:** ^177^Lu-PSMA-617, Pharmacokinetics, Organ dosimetry, Metastatic castration-resistant prostate cancer, Phase II, Biodistribution

## Abstract

**Background:**

^177^Lu-PSMA-617 is a radioligand therapy targeting cells expressing prostate-specific membrane antigen (PSMA). The pharmacokinetics, dosimetry, and safety of ^177^Lu-PSMA-617 in participants with metastatic castration-resistant prostate cancer are described previously. In this study, we describe the blood pharmacokinetics behavior, biodistribution, and dosimetry of ^177^Lu-PSMA-617 to support its clinical use in Chinese participants.

**Results:**

Nine participants were infused with ^177^Lu-PSMA-617 (range: 6985.6–8036.0 MBq). The geometric (Geo)-mean blood terminal half-life was 52.6 h, corresponding to an effective half-life of approximately 40 h. The Geo-mean maximum blood concentration of 11.3 ng/mL was achieved at a median time of 0.217 h post administration. The Geo-mean volume of distribution and clearance were 123 L and 1.62 L/h, respectively. The lacrimal glands received the highest absorbed dose of 3.1 mGy/MBq (Geo-coefficient of variation, 93.7%), followed by the thyroid, kidneys, and salivary glands. The Geo-mean whole-body effective dose was 890 mSv (Geo-coefficient of variation, 112.4%).

**Conclusions:**

The blood pharmacokinetics and organ dosimetry of ^177^Lu-PSMA-617 in Chinese participants with progressive metastatic castration-resistant prostate cancer were consistent with those previously reported. The cumulative absorbed dose, corresponding to six cycles of treatment, was consistent with published literature. This analysis was conducted as part of a phase II study registered as NCT05670106 at ClinicalTrials.gov on November 7, 2022.

## Background

Prostate cancer (PC) is the second most frequent cancer in males globally [1]. During 2022 in China, PC was the seventh leading cause of cancer mortality in males; there were an estimated 134,200 newly diagnosed cases and 47,500 deaths [2]. By 2030, it is estimated that the incidence of PC will have increased by 2.88%, meaning the number of projected deaths would be 81,540 [3]. Compared with the global average, the incidence and mortality rate of PC in China is low; however, incidence has increased by 95.2% over the last 30 years compared with 13.2% across the world [4]. Increased access to screening, aging of the population, and Westernization of diet and lifestyle are potential contributors to this increased incidence rate [4].

Prostate-specific membrane antigen (PSMA) is a transmembrane glycoprotein that is upregulated in poorly differentiated, metastatic, and hormone-refractory cancer cells while being lowly-expressed in other healthy organs [5]. PSMA is more highly expressed in PC with defective DNA repair pathways; this expression increases during PC disease progression and is associated with a higher risk of disease recurrence following curative surgery [6, 7]. Treatment of PSMA+ mCRPC with standard systemic therapies is associated with poor prognoses, indicating a need for more targeted therapies [8].

One strategy to specifically target PSMA-expressing mCRPC is the use of radiolabelled small-molecule PSMA inhibitors [6, 9]. ^177^Lu-PSMA-617 is a β-emitting radionuclide therapy that has demonstrated delivery of high-absorbed doses to tumors with low normal-organ toxicity [9]. A meta-analysis of available efficacy and safety data found that treatment with ^177^Lu-PSMA-617 is an effective therapeutic option with a low toxicity profile in individuals with advanced-stage mCRPC that is refractory to standard therapies [10]. Long-term follow-up data from a phase II study support previous reports of high response rates, low toxicity, and improved quality of life following ^177^Lu-PSMA-617 treatment in participants with progressive PSMA+ mCRPC [11]. In the phase III VISION study, ^177^Lu-PSMA-617 treatment, when added to standard of care, prolonged imaging-based progression-free survival and overall survival in individuals with advanced PSMA+ mCRPC [12].

Dosimetric and pharmacokinetic (PK) characterization of radioligands is crucial to their effective and safe use [13, 14]. These assessments should be done across multiple time points to account for differential organ kinetics and stability in the blood and urine [15, 16]. The PK and dosimetry of ^177^Lu-PSMA-617 has been evaluated in four studies to date [17–20]. These studies showed rapid clearance, and distribution to the salivary glands and kidneys [17–19]. The phase III VISION study included a dosimetry substudy to evaluate ^177^Lu-PSMA-617 treatment exposure levels [20]. Per cycle 1 dosimetry analyses, the lacrimal glands received the highest absorbed dose, followed by the salivary glands, left colon, rectum, and kidneys [20]. Cumulative absorbed doses across six cycles of treatment were estimated from the cycle 1 treatment dose; the extrapolated cumulative absorbed dose was comparable with the observed cumulative doses across the cohort and within individual participants [20].

This study characterizes both the dosimetry and PK following ^177^Lu-PSMA-617 in Chinese participants with progressive PSMA+ mCRPC.

## Methods

### Trial design and interventions

This study was registered as NCT05670106, and evaluated the efficacy, safety, tolerability, PK, and dosimetry of ^177^Lu-PSMA-617 when administered concomitantly with standard of care treatments in Chinese participants with progressive PSMA+ mCRPC. Participants were recruited from study sites across China. Participants received the ^177^Lu-PSMA-617 cycle 1 dose of 7400 MBq (± 10%) by slow intravenous infusion. The objective of this study was to evaluate the consistency of PK and dosimetry results of ^177^Lu-PSMA-617 in Chinese participants with progressive mCRPC compared with previous studies and published literature.

### Participants

Male Chinese participants with histologically, pathologically, and/or cytologically confirmed progressive mCRPC aged ≥ 18 years were included. Participants must also have been ^68^Ga-PSMA-11 positron emission tomography/computed tomography (CT) scan–positive (determined visually based on lesion showing greater uptake intensity compared background liver uptake), and eligible as determined by the central reader. Presence of at least one of the following criteria was used to define progressive mCRPC: serum/plasma prostate-specific antigen progression, soft-tissue progression, and/or progression of bone disease. Participants must have been previously treated with at least one androgen receptor pathway inhibitor and at least one, but no more than two, previous taxane regimens, and must have adequate organ function.

Participants receiving prior PSMA-targeted radioligand therapy were excluded. Additional exclusion criteria include the use of any investigational agents and/or systemic anti-cancer therapy within 28 days prior to enrollment, and the presence of central nervous system metastases and/or current or impending spinal cord compression. Individuals with central nervous system metastases who received therapy and are asymptomatic and neurologically stable without corticosteroids; and individuals with epidural disease, canal disease, and prior cord involvement who have been treated, are stable, and are not neurologically impaired were eligible.

### Pharmacokinetic assessments

Blood samples were collected for each participant from the contralateral arm to the study drug administration at a pre-dose time point, at the end of infusion, and at the following time points following the ^177^Lu-PSMA-617 injection: 20 min (± 5 min), 60 min (± 5 min), 2 h (± 30 min), 4 h (± 30 min), 24 h (± 2 h), 48 h (± 4 h), 72 h (± 6 h), and 168 h (± 12 h). Urine was collected from each participant from the start of the injection through 2 h post injection. Blood samples were immediately processed to measure radioactivity using a gamma counter.

All gamma counters used were pre-validated using a standardized manual provided to the central reader to ensure accuracy and precision. A series standard curve with known ^177^Lu radioactivity, ranging from approximately 0.1 kBq/mL to 700 kBq/mL, was measured in triplicate. A blank sample, containing 0.9% sodium chloride, was also measured for background correction. A centralized pharmacokineticist evaluated the data and performed a linear regression to determine the calibration factor to convert counts per min to radioactivity in Bq. The lower limit of quantification was defined as the lowest activity concentration with an accuracy and precision of ± 20%, and the upper limit of quantification was defined as the highest activity concentration with an accuracy and precision of ± 15%.

Data processing for both whole blood and urine was completed by comparing the gross counts per min from each time point with the lower limit of quantification and the upper limit of quantification specified for the site-specific gamma counter system. Data outside of this range were identified and flagged. Radioactivity data then underwent correction for any background radioactivity, sample dilution, and physical decay of ^177^Lu to determine percent injected activity per mL in whole blood and urine. PK analyses were completed following a non-compartmental analysis of the whole blood radioactivity-based concentration and participant time data using the Plasma (200–202) model type and Linear Trapezoidal Linear Interpolation calculation methods in Phoenix WinNonlin 8.3 (Certara). Urinary drug excretion was determined from the decay-corrected urine concentration and collection volume.

PK analyses were carried out by a centralized pharmacokineticist. Participants with a suitable PK curve for analysis, as assessed by the pharmacokineticist, were included for evaluation.

### Image acquisition

Whole-body planar scans, along with a small imaging reference source of approximately 50–100 MBq, were acquired for each participant at the following time points following the cycle 1 ^177^Lu-PSMA-617 injection: 2 h (1–2 h), 24 h (18–26 h), 48 h (± 12 h), and 168 h (± 12 h). The urine container was imaged alongside the participant at the 2-h time point. Three-dimensional (3D) single-photon emission CT (SPECT) or 3D SPECT/CT scans of the upper abdominal area, covering the kidneys, liver, and spleen, were acquired following the same collection timeline as the whole-body planar scans. Medium-energy collimators were recommended at each site for all scans. A low-dose CT scan was acquired at the 2-h time point.

### Biodistribution and dosimetry assessments

Whole-body planar scans and SPECT images were used for dosimetry assessments. Whole-body planar scans were corrected for scatter photons (C_SC_) using main energy counts (C_main_), lower energy window counts (C_low_), upper energy window counts (C_upper_), and widths in keV of the main energy window (W_main_, 208 KeV with 15% width [± 7.5%]), lower energy window (W_low_, 170 KeV with 15% width [± 7.5%]), and upper energy window (W_upper_, 240 KeV with 10% width [± 5%]) per the equation below:

$$\begin{aligned}\rm C_{SC}&={\rm C_{main}}-\frac{1}{2}\left(\frac{{W}_{main}}{{W}_{low}}\times{C}_{low}+\frac{{W}_{main}}{{W}_{upper}}\times{C}_{upper}\right) \end{aligned}$$

Organ boundaries were drawn manually and adjusted as needed on the whole-body planar images. Threshold-based algorithms were used to determine organ regions of interest (ROIs), which were applied across the imaging time points. Total activity, in MBq, was obtained for each segmented ROI. Whole-body planar scan images were calibrated by manually drawing a boundary around the total body and urine container. A separate boundary around the total body, without the urine container, was also drawn to establish the total body ROI.

The SPECT images were quantitatively reconstructed at site; if this was not possible, SPECT projection data were centrally quantitatively reconstructed using QSPECT software (QSPECT project, Osaka, Japan). The low-dose CT scan was used to build an attenuation map; this was co-registered with an initial reconstruction of the SPECT image using an ordered participant expectation maximization algorithm for attenuation correction and Monte Carlo simulation-based scatter correction. Imaging data were used for dosimetry assessments unless more than one imaging time point was missing or non-evaluable, SPECT images were acquired with the wrong energy window, the low-dose CT attenuation map was missing or incorrect, the SPECT scans did not comprehensively cover all organ activity, or any other quality issues. Quantitatively reconstructed SPECT/CT images were then loaded into the dosimetry software QDOSE^®^ (Telix Pharmaceuticals Limited, North Melbourne, Victoria, Australia).

CT or magnetic resonance imaging scans were acquired during the screening phase, within 21 days prior to enrollment, and were used to determine organ measurements for dosimetry evaluations. Organ volume was determined or verified by a radiologist using low-dose CT scans and the dosimetry software QDOSE^®^. Organ volumes for the kidneys, liver, and spleen were determined from morphological data by segmenting the organ. Two-dimensional boundaries were used to automatically generate 3D boundaries. Threshold-based algorithms were then used to determine volumes. These volumes were applied to all collected SPECT images at all time points. Segmentation was done using automated methods and was adjusted manually as needed. Organ segmentation was performed and/or validated by a radiologist.

Dosimetry analyses were carried out centrally by a medical imaging specialist with experience in dosimetry analysis and were reviewed and approved by an experienced nuclear medicine physician. All imaging data and evaluation results were centrally hosted on an evaluation server. The imaging reference source included in whole-body planar scans was segmented via a drawn ROI boundary, which was applied across all imaging time points. The half-life (t_½_) was calculated using the mono-exponential fitted time activity curve (TAC) of total activity and compared with the theoretical physical t_½_ of ^177^Lu. Any high deviations were clarified with the site, as needed. Red marrow activity (A_RM_) was estimated based on blood activity using the activity concentration in the blood (AC_blood_) in kBq/mL, red marrow to blood concentration ratio (RMBLR; assumed to be one), red marrow mass (m_RM_), and blood density (ƿ_blood_; assumed to be 1.06 g/mL) as described in the equation below [21]:

$${\rm A_{RM} = AC_{blood}\times RMBLR} \times \frac{{m}_{RM}}{{\mathcal{P}}_{blood}}$$

TACs, fitted to a mono- or bi-exponential function, from other source organs were obtained from 3D SPECT images or whole-body planar scans.

Effective t_½_ (t_eff_) was calculated using a determined function fit. Biological t_½_ (t_bio_) was then calculated incorporating the t_½_ (161.6 h) of ^177^Lu. Area-under-the-curve (AUC) measurements were calculated from TAC_fit_ (the mono- or bi-exponential function fit of the TAC) to determine AUC from time of dosing extrapolated to infinity (AUC_inf_) for each organ using the following equation:

$${\rm AUC_{0-inf}}=\:{\int}_{0}^{\infty\:}TA{C}_{fit}dt$$

These measurements were normalized for administered activity to determine time integrated activity coefficients (TIAC) or residence time. TIACs for gastrointestinal organs were determined using the human alimentary tract model [22]. TIACs for the urinary bladder were determined using the voiding bladder model [23]. TIACs for the red marrow were determined using A_RM_ measurements. TIACs in the remaining tissue were calculated by subtracting the TIACs from all source organs from the total body TIACs for all imaging time points.

Dosimetry analysis was carried out using OLINDA/EXM 2.2.3 (Hermes Medical Solutions) by a medical imaging specialist and reviewed by an experienced nuclear medicine physician. Normalized cumulative activity for assessed organs and for the whole body were used for dosimetry calculations. The absorbed dose in mSv and normalized absorbed dose in mSv/MBq were determined for target organs and the whole body. Tissue weighting factors from the International Commission on Radiological Protection Publication 103 were used [24]. The absorbed dose to the lacrimal glands was determined using the spherical model, which assumes a mass of 0.7 g for each gland. S-value calculations were implemented as a continuous interpolation from OLINDA sphere models using power functions.

The absorbed dose measurements from the cycle 1 ^177^Lu-PSMA-617 treatment dose were used to estimate the full dose following six cycles of treatment. This was done by multiplying the cycle 1 absorbed dose by 44.4 GBq, the maximal ^177^Lu-PSMA-617 dose following six cycles of treatment.

All imaging data and evaluations were centrally maintained.

### Study conduct

This study was registered with ClinicalTrials.gov (NCT05670106). Informed consent was obtained from all participants. This study was conducted in accordance with the Declaration of Helsinki and the International Council for Harmonisation of Technical Requirements for Pharmaceuticals for Human Use Good Clinical Practice guidelines. The trial protocol was approved by the Medical Ethics Committees of Fudan University Zhongshan Hospital, Beijing Cancer Hospital, and the First Affiliated Hospital of Guangzhou Medical University, and the Ethics Committees of Sun Yat-sen University Cancer Center and Clinical Trial, and West China Hospital of Sichuan University.

## Results

### Participants

Nine participants received a single dose of ^177^Lu-PSMA-617. The dose ranged from 6985.6 to 8036.0 MBq, which was within the protocol-specified range of 7400 MBq (± 10%). Dosimetry and PK assessments were performed on all nine participants, all of whom completed all the protocol-defined blood PK collections and dosimetric planar and SPECT/CT scans.

### Pharmacokinetics

**Fig. 1:**
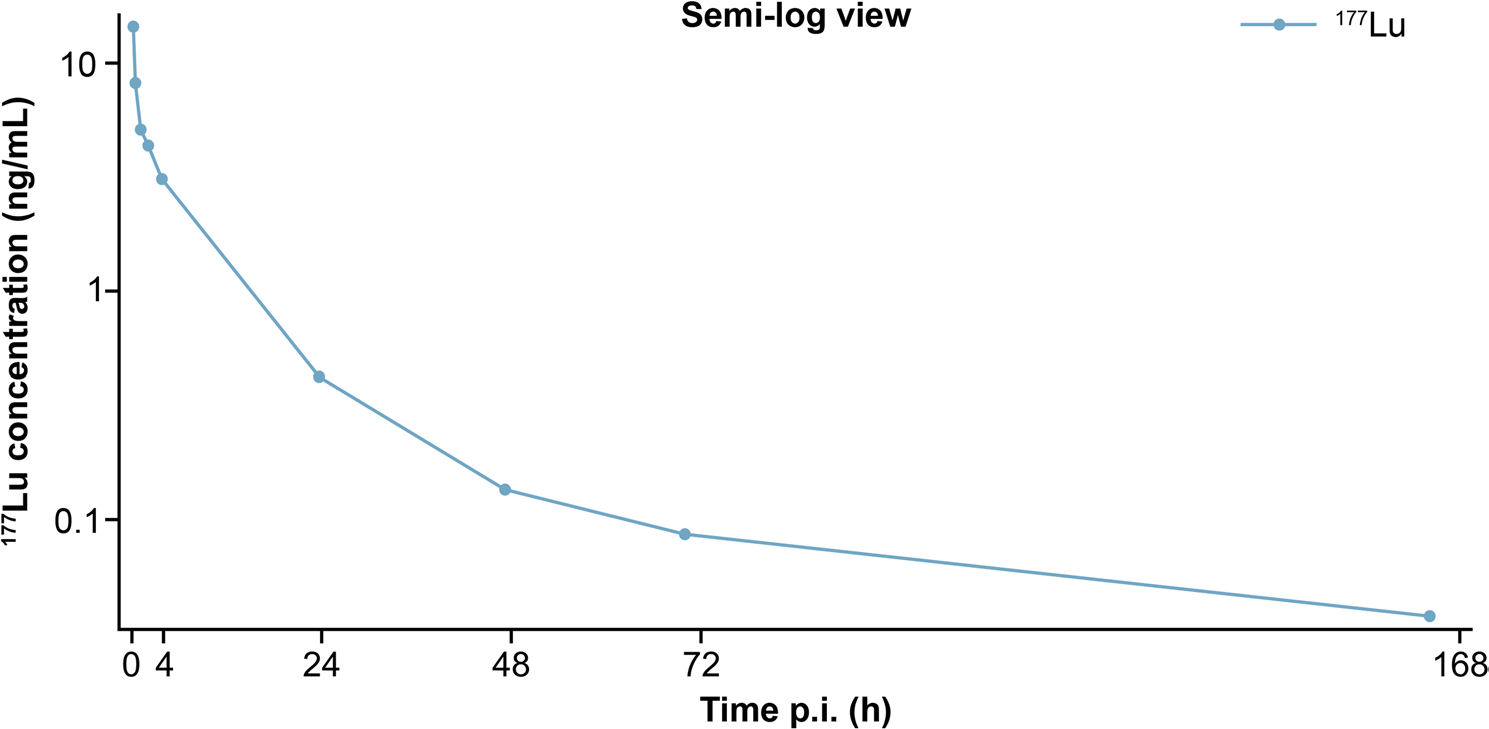
^177^Lu-PSMA-617 whole blood concentration-time profile. ^177^Lu, ^177^Lu-PSMA-617; p.i., post-injection; PSMA, prostate-specific membrane antigen

Blood and urine data were found to be adequate for PK analysis from all nine participants. The whole blood concentration–time profile for ^177^Lu-PSMA-617 was measured for all participants (Fig. 1; Table 1). Geometric (Geo)-mean maximum blood concentration (C_max_) was 11.3 ng/mL, observed at a median time of 0.217 h. Mass-dose-normalized C_max_ (Geo-coefficient of variation [CV]) was 0.103 ng/mL/µg (47.4%). PK analyses determined the Geo-mean (Geo-CV%) volume of distribution based on the terminal phase (V_z_) and the steady-state volume (V_ss_) of 123 L (38.7%) and 34.7 L (78.7%), respectively. Geo-mean (Geo-CV%) AUC from time 0 to the last time point and AUC_inf_ were 64.4 (35.5%) and 68.4 (36.9%) h*ng/mL, respectively. The Geo-mean residence time and Geo-mean terminal t_½_ were 21.4 h and 52.6 h, respectively.

**Table 1:** ^177^Lu-PSMA-617 pharmacokinetic parameters

PK parameter	Our study geo-mean (Geo-CV%)	VISION substudy geo-mean (Geo-CV%) [20]
C_max_, ng/mL (%)	11.3 (53.4)	6.58 (43.5)
T_max_, h (%)	0.241 (40.9)	0.266 (180)
V_z_, L (%)	123 (38.7)	123 (78.1)
V_ss_, L (%)	34.7 (78.7)	NA
AUC_last_, h*%IA/mL (%)	64.4 (35.5)	49.7 (31.6)
AUC_inf_, % (%)	2.96 (150.6)	NA
MRT_inf_, h (%)	21.4 (84.0)	NA
Terminal t_½_, h (%)	52.6 (41.7)	41.6 (68.8)
CL, L/h (%)	1.62 (27.2)	2.04 (31.5)

AUC_inf_, area under curve from time of dosing extrapolated to infinity; AUC_last_, area under curve from time of dosing to the time of last quantifiable concentration; CL, clearance; C_max_, maximum serum concentration; Geo, geometric; h, hours; IA, injected activity; MRT_inf_, mean residence time; NA, not available; PK, pharmacokinetic; PSMA, prostate-specific membrane antigen; t_½_, half-life; T_max_, time to C_max_; Vz, terminal phase volume of distribution; Vss, steady-state volume of distribution

### Dosimetry

**Fig. 2:**
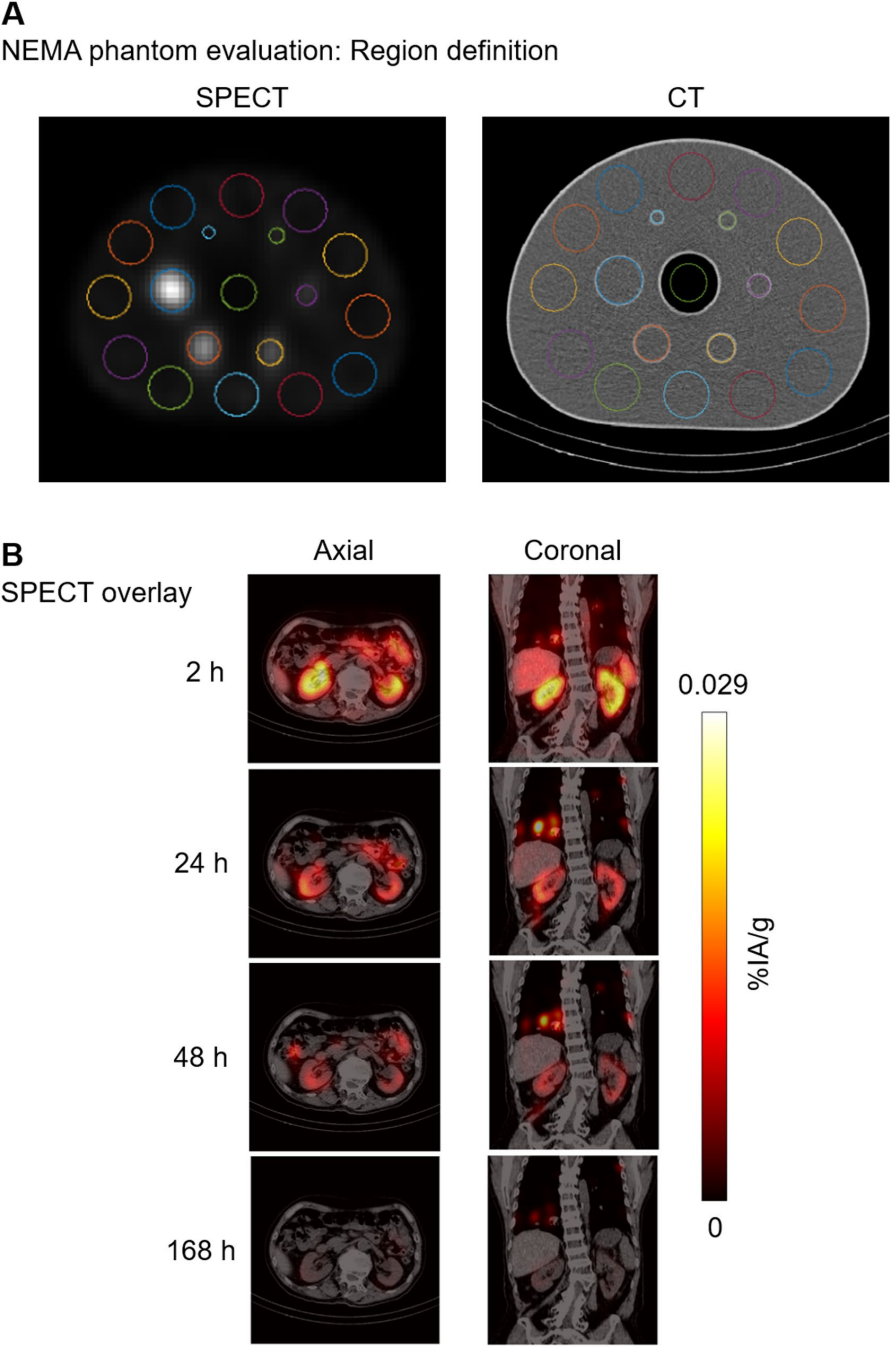
^177^Lu-PSMA-617 dosimetry imaging. (**A**) NEMA phantom evaluation: region definition and (**B**) SPECT/CT overlay. CT, computed tomography; NEMA, National Electrical Manufacturer’s Association; PSMA, prostate-specific membrane antigen; SPECT, single-photon emission computed tomography

**Fig. 3:**
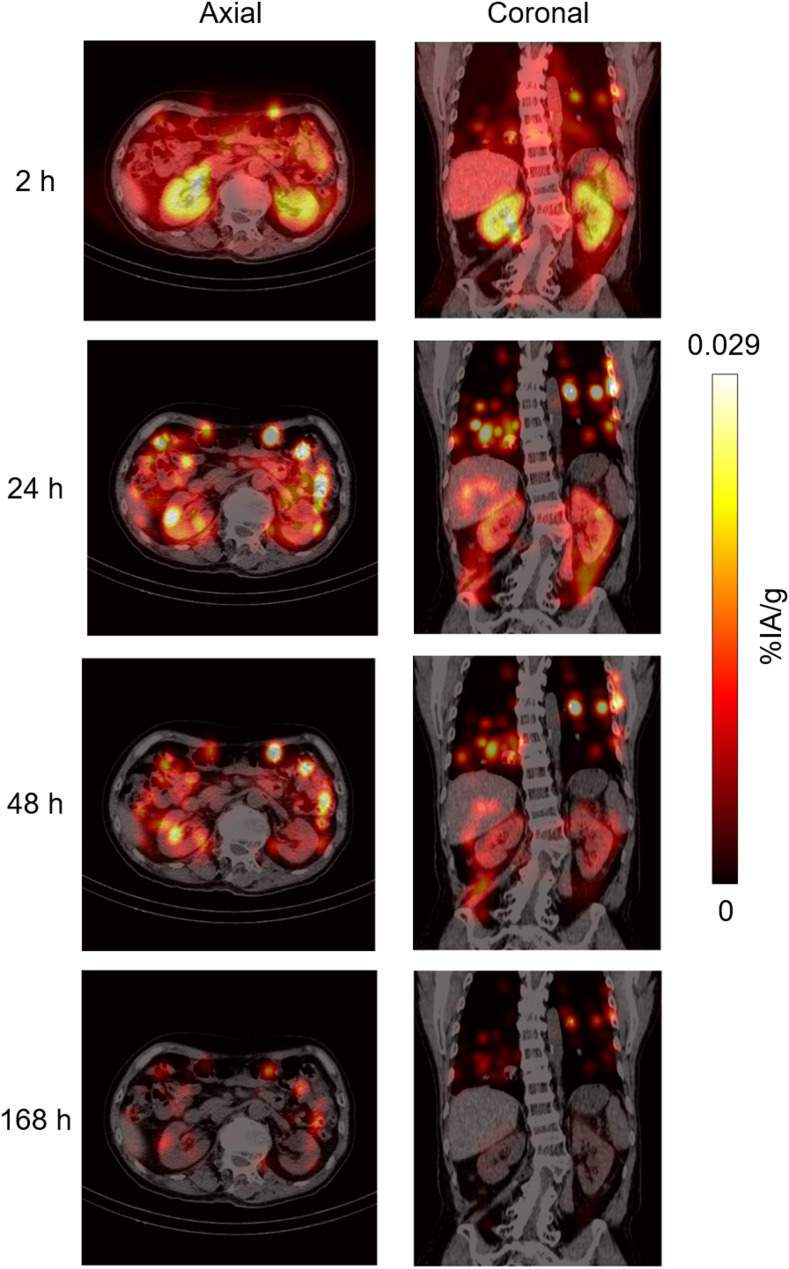
^177^Lu-PSMA-617 dosimetry imaging maximum intensity projection. PSMA, prostate-specific membrane antigen

Whole-body planar scans and 3D SPECT/CT imaging data from the ^177^Lu-PSMA-617 cycle 1 treatment were complete in all nine participants. Representative National Electrical Manufacturer’s Association phantom evaluations (Fig. 2A), SPECT/CT overlay images (Fig. 2B), and maximum ^177^Lu-PSMA-617 intensity projections (Fig. 3) are shown in Figs. 2 and 3.

**Fig. 4:**
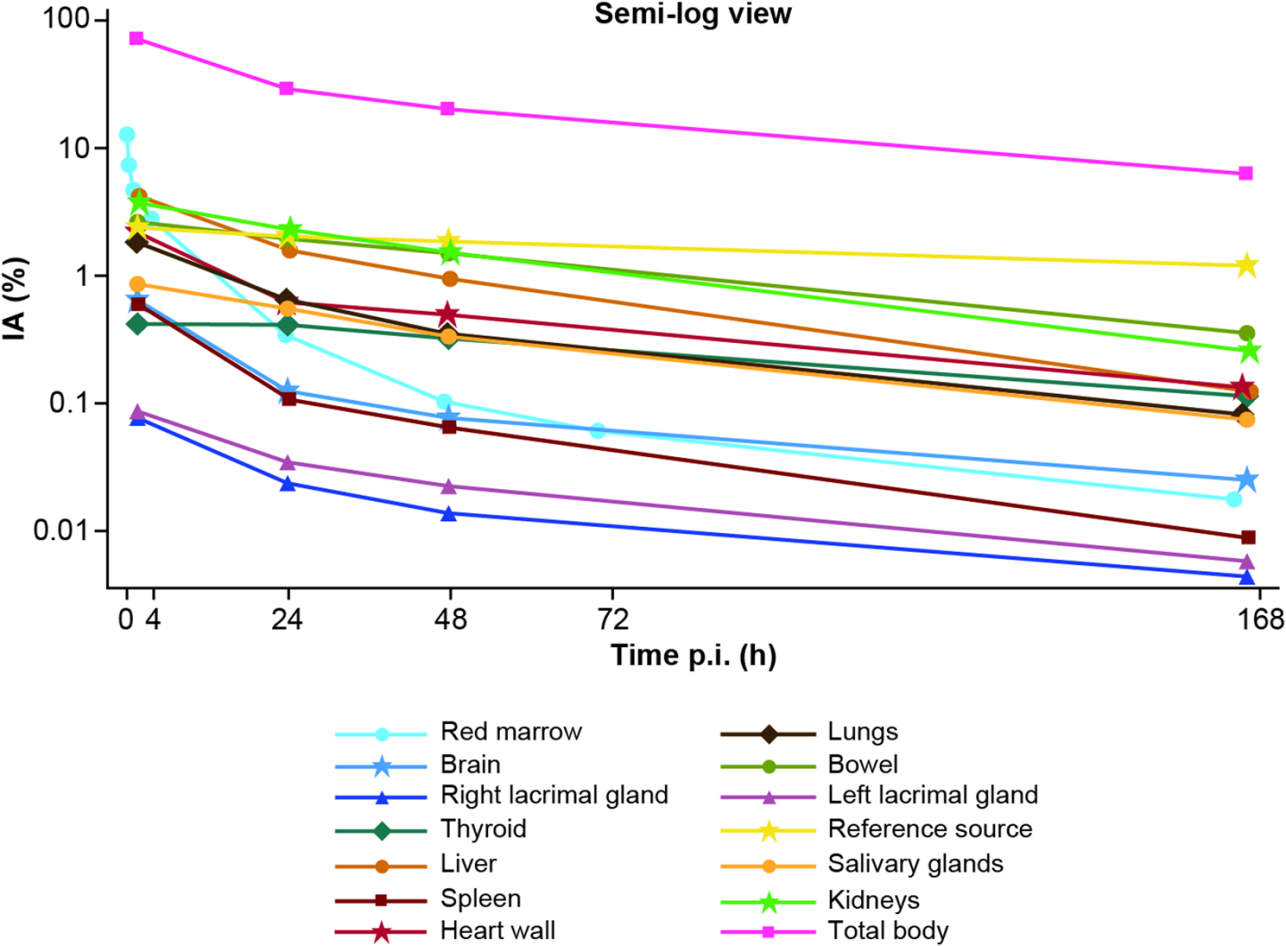
Mean organ % ^177^Lu-PSMA-617 injected activity versus mean time (PK analysis set). IA, injected activity; p.i., post-injection; PK, pharmacokinetic; PSMA, prostate-specific membrane antigen

At the end of the ^177^Lu-PSMA-617 infusion, 13 ± 3.9% of the IA was measured in the red marrow, on average (Fig. 4). By 2 h post infusion, the activity in the red marrow decreased to 4.0 ± 0.89% and uptake in the other analyzed organs increased (Table 2). Overall, the mean total body TAC demonstrated an initial phase of rapid clearance of ^177^Lu-PSMA-617 in the body within the first few hours after infusion (Fig. 4).

**Table 2:** Organ uptake at the 2-h post-injection time point for ^177^Lu-PSMA-617 imaging

Organ	Mean (SD) injected activity, %
Liver	4.2 (1.6)
Red marrow	4.0 (0.89)
Kidneys	3.7 (0.63)
Bowel	2.6 (0.70)
Heart wall	2.2 (0.89)
Lungs	1.8 (1.5)
Salivary glands	0.86 (0.42)
Brain	0.64 (0.20)
Parotid glands	0.60 (0.33)
Spleen	0.59 (0.29)
Thyroid	0.42 (0.61)
Left lacrimal gland	0.087 (0.040)
Right lacrimal gland	0.077 (0.042)

h, hour; PSMA, prostate-specific membrane antigen; SD, standard deviation

^177^Lu-PSMA-617 kinetics in the total body exhibited a bi-exponential trend, with a fast-phase t_eff_ of 7.5 h and a slow-phase t_eff_ of 73 h (Table 3). The corresponding t_bio_ were 8.1 h and 160 h, respectively. Bi-exponential models best fit the kinetics in the kidneys, salivary glands, and thyroid as well. The fast-phase t_eff_ for the kidneys, salivary glands, and thyroid were 7.0 h, 14.0 h, and 5.7 h, respectively, with slow-phase t_eff_ of 43 h, 60 h, and 62 h, respectively. ^177^Lu-PSMA-617 kinetics in the red marrow were best described in three distinct phases, with two fast phases and one slower terminal phase; the t_eff_ for the three phases were 0.31 h, 4.7 h, and 35 h. TIAC measurements from the total body and analyzed organs were then used to determine the residence time (Table 4). The bowel had the highest ^177^Lu-PSMA-617 Geo-mean TIAC of 2.0 MBq*h/MBq, followed by the kidneys (1.8 MBq*h/MBq) and liver (1.2 MBq*h/MBq). In the whole body, TIAC was calculated to be 32 MBq*h/MBq.

**Table 3:** ^177^Lu-PSMA-617 effective and biological half-lives in the total body and analyzed organs

Organ	Mean (SD) t_eff_, h
Red marrow	35 (26)
	4.7 (2.4)
	0.31 (0.28)
Kidneys	43 (20)
	7.0 (5.7)
Salivary glands	60 (41)
	14 (7.0)
Thyroid	62 (18)
	5.7 (4.9)
Total body	73 (22)
	7.5 (6.3)

PSMA, prostate-specific membrane antigen; SD, standard deviation; t_½_, half-life; t_bio_, biological half-life; t_eff_, effective half-life

**Table 4:** TIAC for organs of interest following ^177^Lu-PSMA-617 imaging

Organ	Geo-mean (Geo-CV%) TIAC, MBq*h/MBq (%)
Bowel	2.0 (51.8)
Kidneys	1.8 (65.1)
Liver	1.2 (67.7)
Heart wall	0.74 (60.9)
Lungs	0.50 (86.6)
Red marrow	0.45 (20.9)
Thyroid	0.13 (357.4)
Total body	32 (66.8)

CV, coefficient of variation; Geo, geometric; h, hour; MBq, megabecquerel; PSMA, prostate-specific membrane antigen; TIAC, time integrated activity coefficient

Normalized cumulative activity measurements were used to derive the absorbed dose values for each analyzed organ (Table 5). After infusion of ^177^Lu-PSMA-617, the lacrimal glands were found to receive the highest Geo-mean absorbed dose of 3.1 mGy/MBq, followed by the thyroid (0.71 mGy/MBq) and kidneys (0.56 mGy/MBq) (Table 5). The Geo-mean effective dose across the entire study cohort was 890 mSv.

**Table 5:** Normalized absorbed doses for analyzed organs following cycle 1 ^177^Lu-PSMA-617 treatment

Organ	Our study Geo-mean (Geo-CV%) absorbed dose, mGy/MBq (%)	VISION substudy [20] Mean absorbed dose (SD), Gy/GBq
Lacrimal glands	3.1 (93.7)	2.1 (0.47)
Thyroid	0.71 (378.9)	0.26 (0.37)
Kidneys	0.56 (50.3)	0.43 (0.16)
Salivary glands	0.55 (63.5)	0.63 (0.36)
Urinary bladder wall	0.26 (24.9)	0.32 (0.03)
Rectum	0.20 (34.1)	0.56 (0.14)
Left colon	0.20 (33.8)	0.58 (0.14)
Heart wall	0.19 (74.2)	0.17 (0.12)
Right colon	0.12 (40.3)	0.32 (0.08)
Red marrow	0.044 (47.9)	0.035 (0.020)

CV, coefficient of variation; GBq, gigabecquerel; Geo, geometric; Gy, gray; MBq, megabecquerel; mGy, megagray; PSMA, prostate-specific membrane antigen; SD, standard deviation

Absorbed dose measurements following the cycle 1 treatment dose of ^177^Lu-PSMA-617 were used to extrapolate organ uptake following the six cycles (Table 6).

**Table 6:** Dose estimates following cumulative exposure to ^177^Lu-PSMA-617 treatment

Organ	Our study Estimated absorbed dose, Gy	VISION substudy [20] Mean absorbed dose (SD), Gy
Lacrimal glands	140	92 (21)
Thyroid	32	11 (16)
Kidneys	25	19 (7.3)
Salivary glands	24	28 (16)
Urinary bladder wall	12	14 (1.1)
Left colon	9.0	26 (6.0)
Rectum	8.9	25 (6.2)
Heart wall	8.4	7.8 (5.2)
Right colon	5.4	14 (3.4)
Red marrow	2.0	1.5 (0.9)

Gy, gray; PSMA, prostate-specific membrane antigen; SD, standard deviation

## Discussion

The PK results from our study in Chinese adult male participants with PSMA+ progressive mCRPC are largely consistent with data from the VISION substudy (Table 1). In the VISION substudy, the time to C_max_ (T_max_) following ^177^Lu-PSMA-617 infusion was approximately 20 min in 30 non-Chinese participants [20]. In our study of nine Chinese participants, following ^177^Lu-PSMA-617 infusion, T_max_ occurred approximately 10–15 min after administration. Given that T_max_ is typically achieved at the first collected time point, it reflects differences in blood collection schedules and not in PK behavior. Whole blood concentrations declined with a Geo-mean terminal t_½_ of 41.6 h in the VISION substudy [20]. After accounting for the physical t_½_ of the Lu-177 radionuclide (~ 161 h), the t_eff_ was calculated to be ~ 33 h [20]. In our study, the Geo-mean terminal t_½_ was 52.6 h, yielding an effective t_eff_ of ~ 40 h. In the VISION substudy, the Geo-mean clearance and V_z_ were determined to be 2.04 L/h and 123 L, respectively [20]. In our study, the Geo-mean clearance and V_z_ were determined to be 1.62 L/h and 123 L, respectively. Altogether, clearance, V_z_, and t_½_ are comparable with results from the VISION substudy. Intersubject variability was moderate for most PK parameters, with Geo-CV% ranging from 30 ~ 50%; intersubject variability was higher for V_ss_, with Geo-CV% values > 70%.

When evaluating ^177^Lu-PSMA-617 radioligand therapy, it is critical to assess organs with known PSMA expression (i.e. lacrimal glands, salivary glands), organs that function in radionuclide elimination (i.e. kidneys), or organs at risk of radiation-related toxicities, such as hemotoxicity (i.e. red marrow) [5, 14, 25]. Organ dosimetry results from this study are consistent with previous reports. The Geo-mean (Geo-CV%) absorbed doses in the red marrow (0.044 mGy/MBq [47.9%]) and kidneys (0.56 mGy/MBq [50.3%]) are consistent with previous reports from Kratochwil et al. (kidneys: 0.75 ± 0.19 mGy/MBq), Kabasakal et al. (kidneys: 0.82 ± 0.25 mGy/MBq), and Yadav et al. (red marrow: 0.048 ± 0.059 mGy/MBq; kidneys: 0.99 ± 0.31 mGy/MBq) [17–19].

Blood activity was used in this study to determine red marrow dose, with the assumption that activity concentrations are the same across both. Red marrow PK follows tri-exponential phases (t_eff_ 0.31 h, 4.7 h, and 35 h), reflecting its quick distribution and elimination at the first and second phases, followed by a slower terminal phase. In contrast, total body PK shows bi-exponential behavior with a fast-phase t_eff_ of 7.5 h, reflecting the quick elimination of activity from the body in the first phase, followed by a much slower terminal phase t_eff_ of 73 h. The latter corresponds a t_bio_ of 160 h, which is close to Lu-177 physical t_½_ (~ 161 h).

Organ dosimetry was also comparable with the VISION substudy (Table 5) [20]. In the VISION substudy, highest uptake was seen in the lacrimal glands, salivary glands, colon, rectum, kidneys, and urinary bladder wall [20]. In our study, the lacrimal glands, thyroid, kidneys, and salivary glands received the highest absorbed dose. Altogether, these results suggest that biodistribution following ^177^Lu-PSMA-617 infusion is similar in both populations. Estimated organ and total body absorbed doses following six cycles of ^177^Lu-PSMA-617 treatment (a maximum dose of 44.4 GBq) were also comparable between this study and the VISION substudy (Table 6). Overall, the organ absorbed dose estimate in the Chinese population was within one standard deviation of the reported mean in the VISION substudy, except for the lacrimal glands and thyroid [20].

Our data are largely consistent with published absorbed dose measurements in other studies and with reported exposure thresholds (Table 6). While reported external beam radiation therapy exposure thresholds for the kidneys range from 23 to 40 Gy, the validity of these limits for radioligand therapy remains unclear [16, 26, 27]. The Geo-mean cumulative renal absorbed dose of 25 Gy in Chinese participants is within this range. The recommended external beam radiation therapy thresholds for parotid and submandibular glands, which collectively make up the salivary glands, are 24–26 Gy and 39 Gy, respectively [28]. However, based on available literature, the extrapolated cumulative absorbed dose to the salivary glands is more variable, with some studies reporting up to 84 Gy [17, 18]. In our study, a cumulative absorbed dose of 24 Gy to the salivary glands is reported. The recommended exposure threshold for the red marrow of 2 Gy, derived from radioactive iodine therapy, may not be valid in the context of radioligand therapy [25, 29]. We estimate that the cumulative absorbed dose in the red marrow is 2 Gy in our study. To avoid dry eye or other retinal complications, the recommended cumulative threshold for the lacrimal glands is 40 Gy [30]. While this threshold was exceeded in both this study and the VISION substudy, there is a high degree of uncertainty in estimating lacrimal gland dosimetry due to its small size and proximity to the body surface, particularly when evaluated via whole-body planar scans [20]. Additionally, despite exceeding the recommended absorbed dose, there were no retinal adverse events reported in the VISION study [12].

This study relied on a centralized pharmacokineticist for PK analyses and a central image review process for dosimetry results, allowing for consistent interpretation of all results. The sample size for this study is comparable to other dosimetric and PK assessments of ^177^Lu-PSMA-617; however, the sample sizes in most of these studies are relatively small [17–20]. While our results generally corroborated findings from the VISION substudy and other literature, the variability in reported absorbed doses may be due to differences in dosimetry imaging methodologies and assessment schedules [17, 19].

There was variation in the absorbed dose to the kidneys, lacrimal glands, and thyroid across the study cohort. Participants 1, 2, and 4 had significantly higher kidney absorbed doses of 1.0 mGy/MBq, 0.76 mGy/MBq, and 1.1 mGy/MBq, respectively, compared with the Geo-mean of 0.56 mGy/MBq. The lacrimal glands in participants 1 (10.9 mGy/MBq) and 2 (6.6 mGy/MBq) also received high doses compared with the Geo-mean of 3.1 mGy/MBq. The Geo-mean absorbed dose to the thyroid (0.71 mGy/MBq) was high compared with results from the VISION substudy (0.26 Gy/GBq) [20]. Participants 1 and 8 had considerably high thyroid absorbed doses of 13.5 mGy/MBq and 10.0 mGy/MBq, respectively. Both participants presented with multiple bone metastases. Therefore, activity in the bone and spine posterior to the thyroid could have contributed to perceived thyroid activity in planar images.

## Conclusions

Altogether, these results are consistent with previous PK and dosimetry studies following ^177^Lu-PSMA-617 infusion. Dosimetry following cycle 1 ^177^Lu-PSMA-617 treatment and extrapolated cumulative exposure are largely consistent with previous reports and fall within recommended thresholds. These results support the use of ^177^Lu-PSMA-617 for radioligand therapy in Chinese patients with progressive PSMA+ mCRPC owing to its rapid elimination and high metabolic stability.

## Data Availability

The datasets generated and/or analyzed during this study are not publicly available; the main study is still ongoing, and the primary data have not yet been released.

## Abbreviations

3D: Three-dimensional
AC_blood_: Blood activity concentration
A_RM_: Red marrow activity
AUC: Area under the curve
AUC_inf_: Area under the curve extrapolated to infinity
AUC_last_: Area under the curve from time 0 to the last time point
CL: Clearance
C_low_: Lower energy window counts
C_main_: Main energy counts
C_max_: Maximum concentration
C_sc_: Scatter photon correction
CT: Computed tomography
C_upper_: Upper window counts
CV: Coefficient of variation
GBq: Gigabecquerel
Geo: Geometric
Gy: Gray
h: Hour
IA: Injected activity
Max: Maximum
MBq: Megabecquerel
mCRPC: Metastatic castration-resistant prostate cancer
mGy: Megagray
min: Minimum
m_RM_: Red marrow mass
MRT_inf_: Mean residence time
mSv: Millisievert
NEMA: National Electrical Manufacturer’s Association
ƿ_blood_: Blood density
PC: Prostate cancer
p.i: Post-injection
PK: Pharmacokinetics
PSMA: Prostate-specific membrane antigen
RMBLR: Red marrow to blood concentration ratio
ROI: Region of interest
SD: Standard deviation
SPECT: Single-photon emission computed tomography
t_½_: Half-life
TAC: Time-activity curve
TAC_fit_: Mono- or bi-exponential fit to the TAC
TB: Total body
t_bio_: Biological half-life
t_eff_: Effective half-life
TIAC: Time integrated activity coefficient
T_max_: Time to C_max_
V_ss_: Steady-state volume of distribution
V_z_: Terminal phase volume of distribution
W_low_: Lower energy window width
W_main_: Main energy window width
wo: Without
W_upper_: Upper energy window width
